# Co-occurrence of mutations in *KIF7* and *KIAA0556* in Joubert syndrome with ocular coloboma, pituitary malformation and growth hormone deficiency: a case report and literature review

**DOI:** 10.1186/s12887-020-2019-0

**Published:** 2020-03-12

**Authors:** Marcello Niceta, Maria Lisa Dentici, Andrea Ciolfi, Romana Marini, Sabina Barresi, Francesca Romana Lepri, Antonio Novelli, Enrico Bertini, Marco Cappa, Maria Cristina Digilio, Bruno Dallapiccola, Marco Tartaglia

**Affiliations:** 1 Genetics and Rare Diseases Research Division, Ospedale Pediatrico Bambino Gesù, IRCCS, Viale di San Paolo 15, 00146 Rome, Italy; 2 Unit of Endocrinology, Academic Department of Pediatrics, Ospedale Pediatrico Bambino Gesù, Rome, Italy

**Keywords:** Joubert syndrome, Growth hormone deficiency, Pituitary gland malformation, Oligogenic inheritance

## Abstract

**Background:**

Joubert syndrome is a recessive neurodevelopmental disorder characterized by clinical and genetic heterogeneity. Clinical hallmarks include hypotonia, ataxia, facial dysmorphism, abnormal eye movement, irregular breathing pattern cognitive impairment and, the molar tooth sign is the pathognomonic midbrain-hindbrain malformation on magnetic resonance imaging. The disorder is predominantly caused by biallelic mutations in more than 30 genes encoding proteins with a pivotal role in morphology and function of the primary cilium. Oligogenic inheritance or occurrence of genetic modifiers has been suggested to contribute to the variability of the clinical phenotype. We report on a family with peculiar clinical spectrum Joubert syndrome molecularly and clinically dissecting a complex phenotype, in which hypogonadism, pituitary malformation and growth hormone deficiency occur as major features.

**Case presentation:**

A 7 year-old male was enrolled in a dedicated “Undiagnosed Patients Program” for a peculiar form of Joubert syndrome complicated by iris and retinochoroidal coloboma, hypogonadism pituitary malformation, and growth hormone deficiency. The molecular basis of the complex phenotype was investigated by whole exome sequencing. The concomitant occurrence of homozygosity for mutations in *KIF7* and *KIAA0556* was identified, and the assessment of major clinical features associated with mutations in these two genes provided evidence that these two independent events represent the cause underlying the complexity of the present clinical phenotype.

**Conclusion:**

Beside the clinical variability of Joubert syndrome, co-occurrence of mutations in ciliopathy-associated genes may contribute to increase the clinical complexity of the trait.

## Background

Joubert syndrome (JBTS, MIM PS213300) constitutes a clinically heterogeneous group of developmental delay/multiple congenital anomalies disorders [1]. Major clinical features include hypotonia, ataxia, developmental delay (DD), intellectual disability (ID), oculomotor apraxia, recurrent hyperventilation, and the recognizable molar tooth sign (MTS), which is the obligatory radiological hallmark of the condition. Additional findings include retinal dystrophy, nephronophthisis, hepatic fibrosis and polydactyly, with both inter- and intra-familial variability [2]. JBTS belongs to the ciliopathy spectrum that recognizes defects in genes codifying proteins of the primary cilium and/or basal body and centrosome, where they play a role in formation, structure and function of these organelles. Defective primary cilium is known to impair the cellular chemo- and mechano-sensation as well as signaling, with WNT, SHH and PDGF representing the major involved signaling pathways [3]. These disorders are generally single gene traits, predominantly following recessive inheritance involving more of 30 genes [4]. Biallelic mutations in *KIF7* (MIM# 611254) have unfrequently been reported in a clinically variable group of ciliopathies (hydrolethalus syndromes, HLS, MIM# 614120; acrocallosal syndrome, ACLS, and Joubert-12, JBTS12, MIM# 200990; Al-Gazali-Bakalinova syndrome, MIM# 607131). Homozygosity for truncating mutations in *KIAA0556* (MIM# 616650), another cilium-associated gene, has recently been identified in a few families with JBTS-related phenotype (Joubert syndrome 26, JBTS26, MIM#616784).

The classification from digenic diseases database (DIDA) distinguishes the ‘true digenic’ event (i.e., variants in two loci that are required for expression of the disease, with none of the variants alone displaying a phenotype), from the “genetic modifier” (GM) effect (i.e., two variants segregating independently and resulting in a combination of two distinctive phenotypes) [5]. Oligogenic inheritance or occurrence of genetic modifiers involving variation in a subset of ciliopathy-related genes (e.g. *AHI1*, *CEP41*, *CC2D2A*, *TMEM67*, *KIAA0556* and *KIF7*) has been suggested to contribute to phenotype worsening [6–8]. The extensive use of second generation sequencing techniques makes today possible to assess of the relevance of co-occurrence of variants in ciliopathy-related genes underlying JBTS.

Here, we report the concomitant occurrence of biallelic mutations in *KIF7* and *KIAA0556*, two genes encoding proteins individually implicated in ciliopathies, in a subject with a distinctive JBTS phenotype complicated by ocular coloboma, hypogonadism, pituitary dysgenesis, and growth hormone deficiency (GHD).

## Case presentation

### Clinical features

The patient (OPBG_13–16) was the first child of healthy parents without family history of relevant genetic disorders, who declined consanguinity but stemmed from the same small village in Italy. The mother had a previous spontaneous abortion in first trimester of pregnancy. The delivery occurred spontaneously at 40 + 3 weeks of gestation after an unremarkable pregnancy except for a prenatal ultrasound of cleft lip and palate. Birth weight was 3550 g (0.22 SDS), birth length 51 cm (0.2 SDS), head circumference (OFC) 34 cm (− 0.66 SDS) according to Bertino’s charts [9]. Apgar scores were 5–9 at 1–5 min. At birth, physical evaluation disclosed cleft lip and palate, left microphthalmia, micropenis, cryptorchidism, hand and feet brachydactyly, and positional talipes. He also showed transient hypoglycemia and jaundice. Head ultrasound and magnetic resonance imaging (MRI) revealed dysgenesis of the corpus callosum, ectopic posterior pituitary, hypoplasia of cerebellum, and thin left optic nerve. In the first month, neurological examination revealed truncal hypotonia, and ID/DD with difficulties in mastication and deglutition. Ophthalmological inspection disclosed left microphthalmia and coloboma and right oculomotor nerve palsy; visually evoked potential was absent in the left eye. The auditory brainstem response was bilaterally normal, and heart, abdominal and renal ultrasounds were unremarkable. Blood basal hormone levels performed at 9 days of life resulted in the normal ranges (TSH, fT3, fT4, testosterone, LH, ACTH, prolactin). Serologic tests for CMV and toxoplasma were negative. Evaluation at 2 months showed the weight 4820 g (− 0.2 SDS), length 54 cm (− 0.22 SDS), head circumference 39 cm (− 0.15 SDS). Endocrine assessment documented low IGF1 and IGF-BP3 levels (respectively < 25 ng/ml and < 0.5 mcg/ml). During hypoglycemia (47 mg/dl), growth hormone (GH) was 0.37 ng/ml with a normal cortisol rise (20.28 mcg/dl), while ACTH, TSH, fT4 and prolactin resulted in the normal ranges. Treatment with human GH (hGH) was started (0.035 mg/kg/day). At 6 months, weight and length measurements were 6400 g (− 2.5 SD) and 64 cm (− 2.7 DS), respectively, whereas head circumference fitted adequacy the 50th centile (43 cm). ACTH stimulation test (250 mcg) showed normal adrenal function, while LHRH stimulation test induced a blunted gonadotropin response for age (minipuberty period) (basal LH 0.8, peak LH 2.51 mU/ml; basal FSH 1.6, peak FSH 2.09 mU/ml). Topical application of 2.5% dihydrotestosterone gel (0.2 mg daily for 4 weeks) was prescribed with good response (more the 3.5 cm in length). Endocrine re-evaluation at 2 years, documented normal levels of fT4, TSH, ACTH, and cortisol; IGF1 levels were 92 ng/ml (under GH treatment). At 3 years of age, GH retesting was performed after 1.5 months of washout, indicating GHD; GH peak after clonidine stimulation was 0.17 ng/ml (n.v. > 8) while GH peak after GHRH plus arginine was 1.08 ng/ml (n.v. > 20). IGF1 and IGF-BP3 were low (respectively < 25 ng/ml and < 0.5 mcg/ml), while basal blood levels of fT4, TSH, cortisol, ACTH resulted normal. At 6.5 years, length was 116 cm (− 0.7 SDS), weight was 20.6 Kg (− 0.79 SDS), and the height velocity was 2.4 cm/year (− 3.87 SDS) with 0.030 mg/kg/day GH treatment. For this poor response, GH was discontinued.

**Fig. 1:**
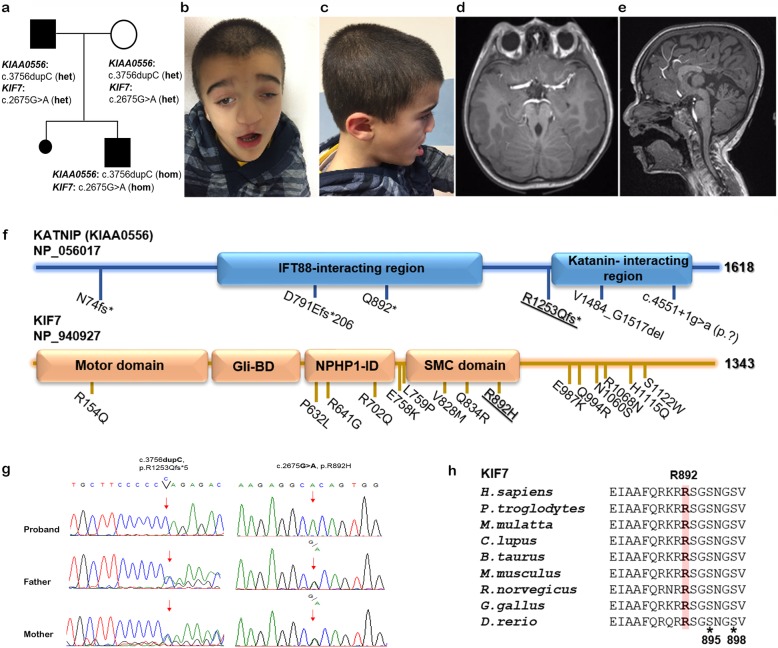
**a** Family tree of the subject with co-occurring mutations in *KIAA0556* and *KIF7*; **b**, **c** Proband’s photos at 7 years; **d**, **e** MRI shows hypoplasia of cerebellar vermis, molar tooth sign (assial, view), and dysgenesis of the corpus callosum (sagittal view); **f** Domain structure of human KIAA0556 (KATNIP) (1618 amino acid residues) and KIF7 (1343 amino acid residues) proteins. The location of the truncating disease-causing variant in KATNIP, and missense change in KIF7 identified is displayed (bold and underlined). Functional domains of KATNIP and KIF7 are also shown. (Abbreviations: Gli-BD, Gli-binding domain; NPHP1-ID, Nephoronophthisis-1-interacting domain; SMC, Structural Maintenance of Chromosomes; **g** Sanger sequencing confirmation of mutations (*KIAA0556*, left panel; *KIF7*, right panel) in proband and his parents. Red asterisk (*) indicates the nucleotide changes; **h** Partial amino acid sequence alignment of the KIF7 SMC domain (NP_940927.2; XP_003314912.2; XP_001094468.1; XP_545852.3; XP_002696621.1 NP_034756.2; 218,828.5; XP_004943897.1; NP_001014816.1). Arginine 892 is highly conserved among vertebrates. Black asterisk (*) indicates phosphorylatable sites, Ser895 and Ser898

At last evaluation (7 years), his height was 116.2 cm (− 1.41 SDS), weight 24.1 Kg (− 0.24 SDS), and his height velocity 0.3 cm/year (− 6.4 SDS) (all SDS were calculated on the Italian Growth Charts) [10]. The stagnation of growth during discontinuation of therapy confirmed GHD. The therapy was hence restarted (0.031 mg/kg/day), and low height velocity was observed. Of note, height velocity could not be precisely calculated due to his spine deformity. Craniofacial dysmorphism (sparse hair, hypertelorism, thick eyebrow, bilateral ptosis, short columella, low-set ears, and signs of cleft lip and palate correction by surgery), flat-overpronated feet with bilateral calcaneovalgus deformity, and severe global ID/DD were confirmed (Fig. 1, panels b and c). He also presented microphthalmia, strabismus, right low vision and left blindness, dysphagia with oral motor dysfunction, and was unable to walk independently. Small retractile gonads and micropenis were also documented. Repeated cerebral MRI confirmed ectopic posterior pituitary and dysgenesis of the corpus callosum, and a small anterior pituitary (maximum height 2 mm), with a thin and partially recognizable stalk. Lateral ventricles were enlarged, particularly in their posterior portions. Both optic nerves and chiasm, and supratentorial deep white matter appeared thinned. Cerebellar vermis hypoplasia was also noticed with high tentorial insertion and large posterior cranial fossa, resulting the classical neuroimaging feature of JBTS, the MTS (Fig. 1, panels d and e). Metabolic analyses for glycosaminoglycan storage and amino acid metabolism disorders ruled out inborn errors of metabolism. Chromosome and high-resolution CGH-array (60 K) analyses excluded the presence of clinically relevant CNVs. Based on its complex phenotype, the proband was enrolled in the Undiagnosed Patients Program at the Bambino Gesù Children’s Hospital for whole exome sequencing (WES) analysis. Collection, use and storage of clinical data, pictures, biological material of the patient and his parents were attained after written informed consent was secured. Clinical and instrumental data of the present subject were consistent with JBTS spectrum; however, the complex phenotype and the occurrence of rare features, including peculiar craniofacial dysmorphism, iris and retinochoroidal coloboma, pituitary malformation, hypogonadism, and GHD, indicated the possibility that the condition might differ from the classical forms of Joubert syndrome (Fig. 1, panels b-e).

### Molecular analyses

WES was performed using genomic DNA extracted from leukocytes of the affected subject and his parents, using the Nextera Rapid Capture Exome kit (Illumina) for library preparation, and a NextSeq500 platform (Illumina) for sequencing. WES data analysis was performed using an in-house implemented pipeline [11–13]. WES details and statistics are described in Supplementary Data 1 and Table S1 (Additional files). Validation of variants and their segregation analyses were assessed by Sanger sequencing.

WES data filtering and prioritization allowed to identify a homozygous missense variant in exon 13 of *KIF7* (c.2675G>A, p.(Arg892His), NM_198525.2) and a homozygous frameshift in exon 19 of *KIAA0556* (c.3756dupC, p.(Arg1253Glnfs*5), NM_015202.2) as the two co-segregating molecular events likely underlying the patient’s condition (Table 1). Heterozygosity in the healthy parents for both variants were confirmed (Fig. 1, panel g). The former variant had previously been reported in public databases (rs143866575; Maximum Allele Frequency, 0.0002213) with uncertain significance based on ACMG classification, but annotated as damaging according to the different in silico predictors (DANN, GERP, MutationAssessor, CADD and MetaDome) (Supplementary Data 1). The latter had not been reported in public and in-house databases. Its functional effect was also predicted to be damaging (CADD score 35), causing premature termination of the protein after five codons.

**Table 1:** Molecular and clinical characteristics of the subject with the occurrence of mutations in *KIF7* and *KIAA0556*

**Subject**	**OPBG_13–16**	
Ethnicity	Caucasian	
Sex	M	
Age (at last evaluation)	7 years	
**Auxological parameter (at birth)**		
Normal growth	Normal	
Length (cm)	51	
Weight (gr)	3550	
OFC (cm)	34	
**Auxological parameter (at last evaluation)**		
Growth delay	Reduced height velocity 0.3 cm/year	
Height (cm)	116.2	
Weight (gr)	24,100	
OFC (cm)	52.5	
**Genetic investigation**		
Karyotype	46,XY	
CGH array	Normal	
Whole Exome Sequencing analysis	See Supplementary data	
**Gene/es**	***KIF7***	***KIAA0556***
Variant in proband (state)	c.2675G>A p.(Arg892His) (homozygous)	c.3756_3757insC, p.(Arg1253Glnfs*5) (homozygous)
Genome region (hg38)	Chr15:89633184C>T	Chr16:27772854dupC
Reference coding isoform	NM_198525.2	NM_015202.2
Reference protein isoform	NP_940927	NP_056017
rsID (dbSNP)	rs143866575	none
Max AF (gnomAD)	T = 0.00022	none
CADD score (PHRED)	29.4	35
MIM#ID	209900	616784
Condition(s)	Joubert syndrome 12, JBTS12 / Acrocallosal syndrome, ACLS	Joubert syndrome 26, JBTS26
Protein	Kinesin-like protein, KIF7	Katanin-interacting protein, KATNIP
Basic function	Cilium-associated protein	Ciliary-base protein
Murine model	*Kif7* knockout mouse model recapitulates major ACLS features	*Kiaa0556* knockout mouse model possess a Joubert syndrome-associated brain-restricted phenotype
**(References)**	[14]	[15]

## Discussion and conclusion

Joubert syndrome is caused by a large repertoire of biallelic variants affecting genes encoding proteins of the primary cilium. Based on the wide inter- and intra-familial phenotypic variability observed in this disorder, the existence of an oligogenic contribution and/or the occurrence of a genetic modifier (GM) effects have been hypothesed [4–8], which has been subsequently confirmed by a recent clinical report [16]. Here, we identify homozygous variants in *KIF7* and *KIAA0556* as the molecular events contributing to a variegated JBTS phenotype associated with clinically relevant endocrine abnormalities.

Systematic revision of the clinical features in previously reported subjects with mutations of *KIF7* and *KIAA0556* confirmed that the complexity of the present phenotype is explained by a model of digenic contribution (Table 2). The co-occurrence of variants in these genes has not been reported thus far. Mutations in *KIF7* have been associated with a broad spectrum of ciliopathies with variable features ranging from lethal hydrocephalus, with or without polydactyly (HLS), to craniofacial dysmorphism, ID, and brain abnormalities with or without MTS (ACLS or JBTS12) [14, 17]. Of note, *KIF7* encodes a member of a family of 14 kinesin motor proteins known to localize from the base to the tip of the cilium governing its structure and length, its architecture and transport [3]. KIF7 protein regulates Gli-Sufu activity thus acting as regulator of hedgehog signaling [18]. The phenotypic variability has been suggested to be the results of a differential impact of individual *KIF7* mutations on protein function [14]. While biallelic truncating mutations causing loss of function of KIF7 have been associated to a more severe phenotype (macrocephaly, ID, facial dysmorphism, polydactyly of the hands/feet, and MTS) [14], biallelic missense variants in *KIF7* have been identified in a few families with variable JBTS spectrum, from classical ACLS phenotype to a clinical presentation (lacking of MTS and having pachygyria), with an overall phenotype different from what was generally associated with inactivating *KIF7* mutations [17, 20]. Notably, endocrine abnormalities including those observed in the present case have never been reported in *KIF7*-associated phenotypes (Table 2).

**Table 2:** Clinical features of the present subject compared with the clinical features individually reported for mutations in *KIF7* and *KIAA0556*

Clinical features	HPO	***KIF7***	***KIAA0556***
Growth delay	HP:0001510	■	■
**Craniofacial**			
Head			
Macrocephaly	HP:0000256	■	■
Wide anterior fontanel	HP:0000260	□	NR
Prominent occiput	HP:0000269	□	NR
Frontal bossing	HP:0002007	■	■
Hypertelorism	HP:0000316	■	■
Nystagmus	HP:0000639	o	■
Ptosis	HP:0000508	o	■
Face			
Prominent forehead	HP:0011220	□	NR
Hypertelorism	HP:0000316	o	o
Short philtrum	HP:0000322	■	o
Midface retrusion	HP:0011800	■	o
Ears			
Malformed ears	HP:0000377	■	o
Preauricular skin tag	HP:0000384	□	NR
Posteriorly rotated ears	HP:0000358	■	o
Low-set, posteriorly rotated ears	HP:0000368	■	o
Eyes			
Microphthalmia	HP:0000568	o	o
Strabismus	HP:0000486	■	o
Thick eyebrow	HP:0000574	o	o
Epicanthal folds	HP:0000286	□	NR
Ptosis	HP:0000508	■	■
Downslanting palpebral fissures	HP:0000494	■	o
Optic atrophy	HP:0000648	■	o
Retinal dystrophy	HP:0000556	□	NR
Nystagmus	HP:0000639	■	■
Coloboma	HP:0000589	■	■
Oculomotor apraxia	HP:0000657	NR	■
Nose			
Hypoplastic nose	HP:0003196	■	■
Anteverted nares	HP:0000463	■	■
Short columella	HP:0002000	o	o
Mouth			
Cleft lip and palate	HP:0008501	■	■
**Cardiovascular**			
Septal defects	HP:0001671	□	NR
Pulmonary valve defects	HP:0005148	□	NR
**Abdomen**			
Abnormality of the kidney	HP:0000077	NR	NR
Abnormality of the liver	HP:0001392	NR	NR
Imperforate anus	HP:0002023	□	NR
Umbilical hernia	HP:0001537	□	NR
Inguinal hernia	HP:0000023	□	□
**Genitourinary**			
Hypospadias	HP:0000047	□	□
Micropenis	HP:0000054	■	■
Cryptorchidism	HP:0000028	■	■
**Skeletal**			
Abnormality of the hand	HP:0001155	□	NR
Abnormality of the foot	HP:0001760	□	NR
**Neurology**			
Global developmental delay	HP:0001263	■	■
Intellectual disability	HP:0001249	■	■
Hypotonia	HP:0001252	■	■
Seizures	HP:0001250	□	□
Cerebellar Ataxia	HP:0001251	□	NR
Neuromuscular		□	□
MRI abnormalities			
Molar tooth sign	HP:0002419	■	■
Hypoplastic or absent corpus callosum	HP:0007370	■	■
Ectopic posterior pituitary	HP:0011747	o	■
Enlarged ventricles	HP:0002119	o	■
Cerebellar hypoplasia	HP:0001321	NR	□
Optic nerve hypoplasia	HP:0000609	■	o
**Endocrine features**			
Panhypopituitarism	HP:0000871	NR	□
Growth hormone deficiency	HP:0000824	o	■
Hypothyroidism	HP:0000821	□	■
**Immunology**			
Recurrent infections	HP:0002719	NR	□
**(References)**		[14, 17–20]	[15, 16, 21, 22]

(■), feature previously reported and present in the proband; (□), feature previously reported but absent in the proband; (o), feature not previously reported but present in the proband; (NR), feature not previously reported and absent in the proband

Biallelic mutations in *KIAA0556* have rarely been reported thus far. To our knowledge, only 4 families with biallelic truncating variants in this gene have been identified and the associated clinical phenotype resembled a form of JBTS spectrum with variable brain malformations including pituitary malformations (Joubert syndrome type 26, JBTS26) [15, 16, 21, 22] (Tables 1 and 2). The first family with three affected subjects of Saudi Arabian origin was described by Sanders et al. [15]. Affected members displayed a homozygous inactivating variant and a mild form of JBTS phenotype including global DD with variable hypotonia, transient tachypnea, variable cerebellar hypoplasia, and MTS. Two out of three affected subjects had panhypopituitarism with hypoplasia/aplasia of the anterior pituitary and an ectopic posterior pituitary. The second reported family included an affected subject having a mild form of JBTS phenotype characterized by hypotonia, DD, ataxia, oculomotor apraxia, nystagmus and bilateral ptosis. Brain abnormalities including thin corpus callosum and MTS had been also documented by MRI scan [21]. The third family had recently been reported by Cauley et al. [16]; remarkably, the affected subjects from consanguineous parents showed the co-inheritance of a frameshift *KIAA0556* mutation and a nonsense variant in a polymicrogyria-associated gene, *ADGRG1*. They display central nervous system malformations including diffuse polymicrogyria, lissencephaly and cerebellar vermis, pons, and brain stem hypoplasia, and MTS. This clinical complexity was explained by the additive effect of the two truncating mutations [16]. More recently, a fourth case with two compound heterozygous variants in *KIAA0556* (c.[2373del]/c.[4551 + 1G-A]) resulting in an abnormal transcript with an in-frame deletion by skipping of exon 25 (102 bp) has been reported [22]. The affected subject showed hypotonia, DD, hypoplastic pituitary, agenesis of corpus callosum, oculomotor apraxia but not coloboma, nystagmus, microphthalmia and craniofacial dysmorphism, as noted in our patient. Interestingly, hypoplasia/aplasia of corpus callosum, cerebellar defects, enlargement of the ventricles, pituitary malformation, and MTS frequently occurred in affected subjects, indicating that defective KIAA0556 likely impacts specifically in brain development and morphology. KIAA0556 (katanin-interacting protein, KATNIP) is evolutionarily conserved, and highly expressed in brain. It is localized to the ciliary base and axoneme where it aids to regulate microtubule dynamics and ciliary integrity. In *C. elegans*, its orthologue interacts with an ARL13B orthologue to control the cilium integrity. Homozygous *Kiaa0556*-null mice show a variable hydrocephalus phenotype with enlargement of the ventricles resulting from a block of cerebrospinal fluid flow in the cerebral aqueduct, confirming a JBTS-associated brain-restricted phenotype [15].

In the present case, homozygosity for *KIAA0556* variant is assumed to have a truncating impact on the mature protein. On the other hand, the change p.(Arg892His) in KIF7 affects the highly conserved Arg892 residue within structural maintenance of chromosomes (SMC) domain [23]. Interestingly, Arg892 is predicted to be intolerant to changes, being localized very close to the phosphorylatable sites Ser895 and Ser898 within a conserved consensus motif, which is thought to be implicated in the regulatory activity of the protein [24] (Fig. 1, panel h). Few missense changes affecting conserved residues into the SMC domain of KIF7 have previously been reported, and established to impair cilia stability and function, hence causing of ACLS [14, 23].

The clinical phenotype in the present case is distinctive and likely caused by the co-occurrence of two ciliopathies (ACLS/JBTS12 and JBTS26) in the same individual. Detailed clinical features are listed in Table 2. Basing on the minimal diagnostic criteria suggested by Courtens et al. [25], features in the present subject are consistent with the diagnosis of ACLS/JBTS12 more than JBTS26, satisfying three out of the four following criteria: total or partial absence of the corpus callosum, craniofacial anomalies, moderate-severe ID and polydactyly. Accordingly with ACL phenotype, craniofacial dysmorphism, including macrocephaly with frontal bossing, hypertelorism, downslanting palpebral fissures, ptosis and strabismus, depressed nasal tip and cleft lip and palate, were also noted (Table 2). Same consideration applies for the central nervous system features, such as profound ID, severe psychomotor delay, and brain malformations. Of note, polydactyly and skeletal defects, considered major features of ACLS, were absent in our patient. Divergent from ACLS/JBTS12, the present subject showed eye abnormalities, including oculomotor apraxia, endocrine disorders such as GHD and specific MRI features (enlargement of the ventricles, high T2 signal in the white matter, reduction of cortical thickness, and pituitary malformation), which have been reported in subjects with mutations in *KIAA0556*. Additional features including microphthalmia, iris and retinochoroidal coloboma, nystagmus, which are rarely seen in both ACLS/JBTS12 and JBTS26, were here present.

In conclusion, our findings document that the complex phenotype occurring in a subset of patient with JBTS can be the result of concomitant mutations in cilium-associated genes. In this specific case, the co-occurrence of two rare/private variants of *KIF7* and *KIAA0556* likely contributes to additional defects of the primary cilium causing congenital organ malformations that are not individually observed in JBTS12 nor in JBTS26. Notably, GM effect of *KIF7* with other cilia-associated *loci* (i.e. *TMEM67*, *CEP41*, and Bardet-Biedl syndrome genes) does not seem to be so infrequent in ciliopathies [14, 19, 26]. Similarity, mutations in *KIAA0056* have previously been documented to co-associate with *ADGRG1* variants contributing to a complex trait resulting in two distinct phenotypes (JBTS26 and BFPP bilateral frontoparietal polymicrogyria, MIM#606854) [16]. This study corroborates previous reports suggesting that the complexity of the JBTS phenotype can be exacerbated by GM occurrences, more commonly than formerly appreciated.

## Supplementary information

**Additional file 1: Supplementary Data 1.** Whole exome sequencing (WES) data analysis was carried out by using an in-house implemented pipeline. Detailed methods and relative references are provided. **Supplementary Table S1.** Whole exome sequencing metrics, statistics and output.

## Data Availability

Not applicable. We are submitting the data together with this manuscript.

## Abbreviations

ACLS: Acrocallosal syndrome
ACMG: American College of Medical Genetics
ACTH: Adrenocorticotropic hormone
*AHI1*: Abelson Helper Integration Site 1
CADD: Combined Annotation Dependent Depletion
*CC2D2A*: Coiled-Coil and C2 Domains-Containing Protein *2A*
*CEP41*: Centrosomal Protein, 41-KD
CMV: Cytomegalovirus
CNVs: Copy number variations
DANN: Deep Learning Approach for Annotating of genetic variants
DD: Developmental delay
DIDA: Digenic diseases database
fT3: Free triiodothyronine
fT4: Free thyroxine
GERP: Genomic Evolutionary Rate Profiling
GHD: Growth hormone deficiency
GM: Genetic modifier
hGH: Human growth hormone
HLS: Hydrolethalus syndromes
ID: Intellectual disability
IGF1: Insulin-like growth factor 1
IGF-BP3: Insulin like growth factor binding protein 3
JBTS: Joubert syndrome
KATNIP: Katanin-interacting protein
KIF7: Kinesin Family Member 7
LH: Luteinizing hormone
MRI: Magnetic resonance imaging
MTS: Molar tooth sign
OFC: Head circumference
PDGF: Platelet-derived growth factor
SDS: Standard deviation scores
SHH: Sonic Hedgehog Homolog
*TMEM67*: Transmembrane Protein 67
TSH: Thyroid-stimulating hormone
WES: Whole exome sequencing
WNT: Wingless-type
