# Supplementary material for: Co-occurrence of mutations in KIF7 and KIAA0556 in Joubert syndrome with ocular coloboma, pituitary malformation and growth hormone deficiency: a case report and literature review
Source: BMC Pediatr. 2020 Mar 12;20:120. doi: 10.1186/s12887-020-2019-0 (PMC7066839; doi:10.1186/s12887-020-2019-0)
Supplement: Supplementary file 1 — Additional file 1: Supplementary Data 1. Whole exome sequencing (WES) data analysis was carried out by using an in-house implemented pipeline. Detailed methods and relative references are provided. Supplementary Table S1. Whole exome sequencing metrics, statistics and output. [file 12887_2020_2019_MOESM1_ESM.pdf]

Additional data

Supplementary Data 1

Whole exome sequencing (WES) analysis was performed on genomic DNA extracted from peripheral blood leukocytes of the affected subject and his parents. Exome capture was carried out using Nextera Rapid Capture Exome Kit V1.2 (Illumina), and the following data analysis was performed using an in-house implemented pipeline which mainly take advantage of the BWA reads aligner (V.0.7.12, Li and Durbin, 2009) and Genome Analysis Toolkit (GATK V.3.7) (McKenna et al., 2010). GATK tools were used for realignment of sequences encompassing INDELs and for base quality recalibration. SNVs and small INDELs were identified by means of the GATK's HaplotypeCaller used in gVCF mode, followed by family-level joint genotyping and phasing. Finally, they were filtered by quality using a hard-filters strategy according to GATK's latest best practices. High-quality variants were then filtered against public databases (dbSNP150 and gnomAD 2.0) to retain private and clinically associated variants, and annotated variants with unknown frequency or having MAF <0.1%, and occurring with a frequency <1% in an *in-house* database including frequency data from approximately 1600 population-matched WES. SnpEff toolbox (V.4.3) was used to predict the functional impact of variants, which were filtered to retain only those located in exons with any effect on the coding sequence, and splice site regions (variants located from −3 to +8 with respect to an exon-intron junction). Functional annotation of variants was performed using SnpEff V.4.3 and dbNSFP V.2.9 (Cingolani et al., 2012; Liu et al., 2013; Kircher et al., 2014; Dong et al., 2015). WES statistics are reported in Supplementary Table 1. Functional impact of variants was analyzed by Combined Annotation Dependent Depletion (CADD) V.1.3, M-CAP V.1.0, InterVar V.0.1.7 and MetaDome algorithms (Kircher et al., 2014; Jagadeesh et al., 2016; Li and Wang, 2017; Wiel et al., 2019), to obtain clinical interpretation according to ACMG/AMP 2015 guidelines (Li and Wang, 2017). Data annotation predicted 19,871 high-quality variants having functional impact (*i.e.*, non-synonymous, indels and splice site changes) in the proband. Among them, 589 private and rare changes were retained for further analyses. Variants were prioritized on the basis of the functional relevance of genes, taking into account both dominant and recessive inheritance models.

References Supplementary Data 1

Li H and Durbin R. Fast and accurate short read alignment with Burrows-Wheeler transform. Bioinformatics. 2009;25:1754-1760.

McKenna A, Hanna M, Banks E, Sivachenko A, Cibulskis K, et al. The Genome Analysis Toolkit: a MapReduce framework for analyzing next-generation DNA sequencing data. Genome Res. 2010;20:297-303.

Cingolani P, Platts A, Wang le L, et al. A program for annotating and predicting the effects of single nucleotide polymorphisms, SnpEff: SNPs in the genome of Drosophila melanogaster strain w1118; iso-2; iso-3. Fly. 2012;6:80-92.

Liu X, Jian X, Boerwinkle E. dbNSFP v2.0: a database of human non-synonymous SNVs and their functional predictions and annotations. Hum Mutat. 2013;34:E2393-402.

Kircher M, Witten DM, Jain P, O'Roak BJ, Cooper GM, Shendure J. A general framework for estimating the relative pathogenicity of human genetic variants. Nat Genet. 2014;46**:**310–5.

Dong C, Wei P, Jian X, Gibbs R, Boerwinkle E, Wang K, Liu X. Comparison and integration of deleteriousness prediction methods for nonsynonymous SNVs in whole exome sequencing studies. Hum Mol Genet. 2015;24**:**2125-37.

Jagadeesh KA, Wenger AM, Berger MJ, Guturu H, Stenson PD, Cooper DN, Bernstein JA, Bejerano G. M-CAP eliminates a majority of variants of uncertain significance in clinical exomes at high sensitivity. Nat Genet. 2016;48:1581-1586.

Li Q, Wang K. InterVar: Clinical Interpretation of Genetic Variants by the 2015 ACMG-AMP Guidelines. Am J Hum Genet. 2017;100:267-280.

Wiel L, Baakman C, Gilissen D, Veltman JA, Vriend G, Gilissen C. MetaDome: Pathogenicity analysis of genetic variants through aggregation of homologous human protein domains. Hum Mutat. 2019;40:1030-1038.

Supplementary Table S1. Whole exome sequencing metrics, statistics and output

	OPBG_13-16
Target regions coverage, 2x[1]	96.9%
Target regions coverage, 10x[1]	95.2%
Target regions coverage, 20x[1]	92.2%
Average sequencing depth on target[1]	95x
Number of variants with predicted functional effect	19,871
Novel, clinically associated, and unknown/low frequency variants[2]	589
Putative disease genes (autosomal recessive/X-linked inheritance)[3]	6[4]
Putative disease genes (*de novo* variants)[3]	0

[1]Referred to Nextera Rapid Capture Exome Kit V1.2 (Illumina).

[2]MAF <0.1% in gnomAD V. 2.0 databases, and frequency <1% in our *in-house* database.

[3]Filtering retained genes with high-quality and functionally relevant variants by excluding those predicted as benign by CADD (scaled score <15) and M-CAP algorithms or benign/likely benign by interVar.

[4]*APEX2* (c.622T>C, p.Trp208Arg), *MAGEA4* (c.799C>T, p.Pro267Ser), ***KIF7* (c.2675G>A, p.Arg892His),** *BFAR* (c.188C>A, p.Ala63Glu), ***KIAA0556* (c.3756dupC, p.Arg1253fs*5),** *OBSCN* (c.5332C>T, p.Gln1778*; c.11362C>A, p.Pro3788Thr).
